# Accessory Gland as a Site for Prothoracicotropic Hormone Controlled Ecdysone Synthesis in Adult Male Insects

**DOI:** 10.1371/journal.pone.0055131

**Published:** 2013-02-01

**Authors:** Julie L. Hentze, Morten E. Moeller, Anne F. Jørgensen, Meghan S. Bengtsson, Anna M. Bordoy, James T. Warren, Lawrence I. Gilbert, Ole Andersen, Kim F. Rewitz

**Affiliations:** 1 Department of Science, Systems and Models, Roskilde University, Roskilde, Denmark; 2 Department of Biology, University of Copenhagen, Copenhagen, Denmark; 3 Department of Biology, The University of North Carolina at Chapel Hill, Chapel Hill, North Carolina, United States of America; U. Kentucky, United States of America

## Abstract

Insect steroid hormones (ecdysteroids) are important for female reproduction in many insect species and are required for the initiation and coordination of vital developmental processes. Ecdysteroids are also important for adult male physiology and behavior, but their exact function and site of synthesis remains unclear, although previous studies suggest that the reproductive system may be their source. We have examined expression profiles of the ecdysteroidogenic Halloween genes, during development and in adults of the flour beetle *Tribolium castaneum*. Genes required for the biosynthesis of ecdysone (E), the precursor of the molting hormone 20-hydroxyecdysone (20E), are expressed in the tubular accessory glands (TAGs) of adult males. In contrast, expression of the gene encoding the enzyme mediating 20E synthesis was detected in the ovaries of females. Further, Spookiest (Spot), an enzyme presumably required for endowing tissues with competence to produce ecdysteroids, is male specific and predominantly expressed in the TAGs. We also show that prothoracicotropic hormone (PTTH), a regulator of E synthesis during larval development, regulates ecdysteroid levels in the adult stage in *Drosophila melanogaster* and the gene for its receptor Torso seems to be expressed specifically in the accessory glands of males. The composite results suggest strongly that the accessory glands of adult male insects are the main source of E, but not 20E. The finding of a possible male-specific source of E raises the possibility that E and 20E have sex-specific roles analogous to the vertebrate sex steroids, where males produce primarily testosterone, the precursor of estradiol. Furthermore this study provides the first evidence that PTTH regulates ecdysteroid synthesis in the adult stage and could explain the original finding that some adult insects are a rich source of PTTH.

## Introduction

Ecdysteroids are insect steroid hormones that elicit and coordinate the molting cycle during larval-pupal-adult development [1]–[3]. They are produced as ecdysone (E) in the prothoracic gland (PG) and further metabolized to the principal molting hormone, 20-hydroxyecdysone (20E), in target tissues [2]–[4]. In the larval stages, molting and metamorphosis are initiated by pulses of ecdysteroids, whose synthesis is stimulated by the release of prothoracicotropic hormone (PTTH) from the brain. PTTH in turn activates E synthesis in the PG through its receptor Torso [5]. Ecdysteroids are synthesized from cholesterol (C) by a series of reactions primarily mediated by cytochrome P450 (P450) enzymes encoded by a group of genes known as the Halloween genes [2]. The initial catalytic conversion of C to 7-dehydrocholesterol (7dC) requires a Rieske oxygenase called Neverland [6], [7]. Although the following possibly rate-limiting Black Box oxidation of 7dC to the 5β-ketodiol is incompletely understood, it is known to involve the action of at least two enzymes, the dehydrogenase Shroud and Spook (Spo) [8], [9]. The final three reactions that convert the 5β-ketodiol to E are mediated by Phantom (Phm), Disembodied (Dib) and Shadow (Sad), all encoded by *P450* genes [10]–[13]. E produced and released from the PG is converted to 20E in target tissues by another P450 enzyme, Shade (Shd) [14]. These Halloween P450 enzymes have been structurally conserved in arthropods and orthologs are found in the genome of insects and even the crustacean water flea *Daphnia pulex* [15]. It is believed that orthologs from different species have the same function, although functional conservation has not been demonstrated in all insects [15]. Surprisingly, the most structurally conserved of these steroidogenic P450 enzymes, Spo, an enzyme believed to function in the rate-limiting Black Box conversion, has not been conserved as a single ortholog [8], [15] (Figure S1). *Drosophila* carries two paralogs of this gene, *spook* (*spo*; *Cyp307a1*) and *spookier* (*spok*; *Cyp307a2*) whereas lepidopterans seems to have a single ortholog, *spo* [8]. In the honey bee, *Apis mellifera*, a single ortholog, *spookiest* (*spot*; *CYP307B1*) exists. *spo* and *spok* show about 57% primary structure similarity and encode P450 enzymes with the same function [8], [15], [16]. In *Drosophila*, *spo* is expressed during embryonic development and in the adult female ovaries, but not in the larval PG. Expression in the larval PG is occupied by *spok* to support ecdysteroidogenesis during postembryonic development. Thus, these genes provide the same function in distinct tissues at different times during development [8]. Although it has not been demonstrated that Spot has the same function as Spo and Spok, the fact that it is a member of the highly conserved CYP307 family strongly suggest that it is functionally conserved. In support of this, *spot* is the only *spo*-like gene found in *Apis* [15].

During *Drosophila* larval growth the Halloween genes are expressed in the PG cells of the ring gland [4]. Adult insects of both sexes contain ecdysteroids, although the PG degenerates during metamorphosis [17], [18], and thus ecdysteroids must be synthesized elsewhere in adult insects. In adult female *Drosophila, spo, phm, dib, sad* and *shd* are expressed in the ovaries, the site of ecdysteroid biosynthesis in females [10]–[14]. Ecdysteroids synthesized in the ovaries are required for vitellogenesis and normal oogenesis in *Drosophila* [19]–[21]. For example, female *spo* mutants rescued to adulthood, which presumably are incapable of producing 20E, are sterile [8]. Although adult males are known to produce ecdysteroids, little is known about the site of synthesis and their role in males [22]. Some studies suggest that ecdysteroid synthesis occurs in the reproductive system of adult males [23], [24], and a recent study shows that the Halloween genes are expressed in the male accessory glands of the mosquito *Anopheles gambiae* that likely synthesizes ecdysteroids *de novo* [25].

Classical biochemical studies have shown that several non-ecdysteroidogenic tissues have the capacity to mediate the final three hydroxylations in the production of E [26], [27], which can be explained by some basal expression of *phm, dib* and *sad* observed in multiple tissues [28]. On the other hand, *de novo* E production from C is limited to the PG cells during larval life, presumable because the other tissues lack one or more factors required to endow a tissue with the biosynthetic capacity to mediate the upstream reactions of the Black Box. Enzymes in the CYP307 family match the criteria for such a factor.

We chose to analyze the expression of the Halloween genes, including the two *CYP307* family paralogs, *spo* and *spot*, to investigate their role and possible sites of ecdysteroidogenesis in the flour beetle *Tribolium castaneum.* So far expression of a *CYP307B* family gene has not been reported in any insect species. We show here that *spot* expression is male-specific and primarily localizes to the tubular accessory glands (TAGs) of the reproductive system in *Tribolium*. Expression of *phm*, *dib* and *sad*, but not *shd*, was also detected in the TAGs. We also show data indicating that *torso* is expressed in the male accessory glands of *Drosophila* and demonstrate that PTTH, the Torso ligand that stimulates E synthesis during development, has a similar function in adults. The results suggest that the TAGs, like the larval PG, have the biosynthetic complement to produce E, but not 20E, possibly under control of PTTH. Thus, the male and female reproductive systems of *Tribolium* may have distinct capacities for producing E and 20E.

## Results

### Expression of the Steroidogenic P450 Enzymes in *Tribolium*

**Figure 1:**
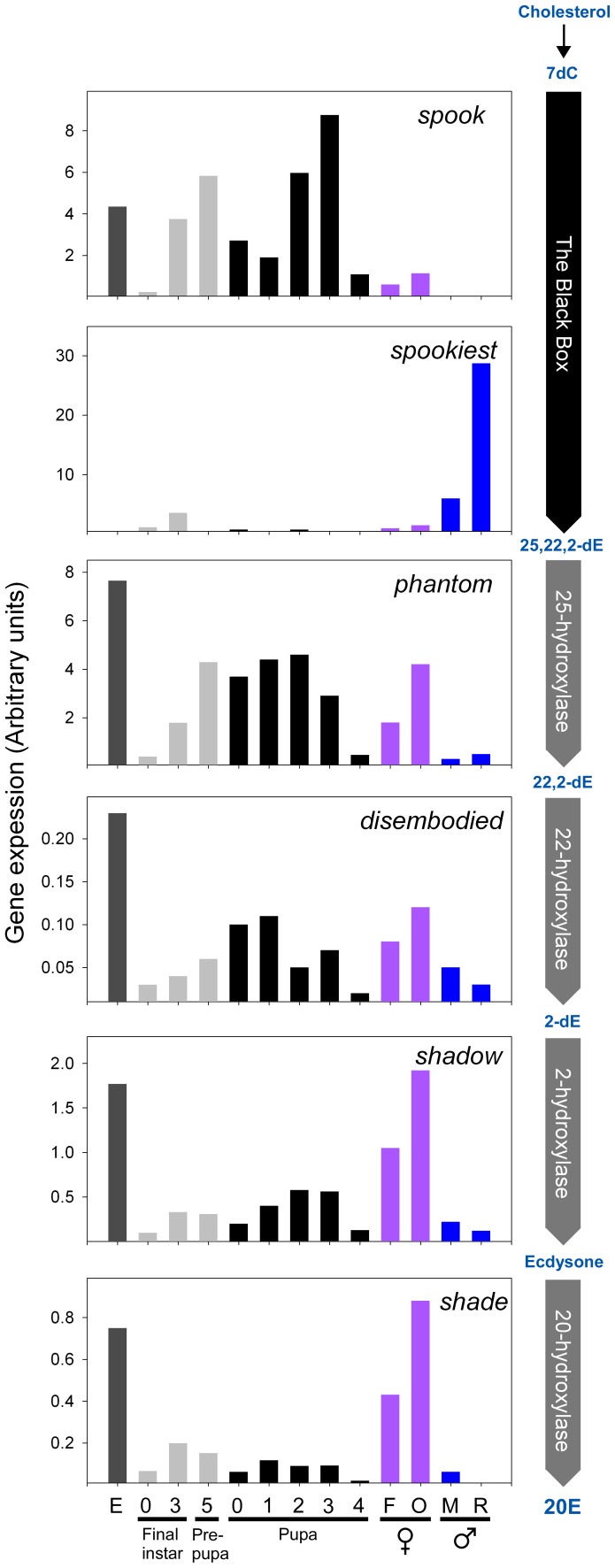
Halloween gene expression during development and in adult *Tribolium*. The relative mRNA distribution of the Halloween genes during development and in adults was measured by qPCR. Expression was normalized to *rpS3* mRNA levels. The step in the ecdysteroid biosynthetic pathway catalyzed by each enzyme is shown on the right. Numbers on the x-axis indicate the day of each stage. E; embryo, F; females, O; ovaries, M; males, R; reproductive systems of males, 7dC; 7-dehydrocholesterol, 2,22,25-dE; 2,22,25-trideoxyecdysone (ketodiol), 2,22-dE; 2,22-dideoxyecdysone (ketotriol), 2-dE; 2-deoxyecdysone, 20E; 20-hydroxyecdysone. Note that the Black Box is believed to include multiple uncharacterized reactions converting 7dC to the ketodiol.

Expression of *spo*, *spot*, *phm*, *dib*, *sad* and *shd* was measured in embryos, final instar larvae, pupae and in adult *Tribolium* (Figure 1). In adults expression was measured in the reproductive system and in whole animals of both sexes. During development, expression of *spot* was below reliable detection levels, even using sensitive quantitative PCR (qPCR). However, for the other Halloween genes examined here, expression was observed during all stages of development analyzed. Expression of *spo*, *phm*, *dib*, *sad* and *shd* was high in the embryo and increased during the final larval instar. In the pupal-adult stage, expression of these genes was detected from day 0–4 while in adult females expression of all genes, except *spot*, was observed with higher expression in the ovaries compared to whole female animals. This shows that *spo*, *phm*, *dib*, *sad* and *shd* are expressed mainly in the ovaries in adult females.

Most of the Halloween genes also showed expression in adult males, where *spo* expression was very low and at the limit of detection. *Spot*, on the other hand, was expressed exclusively in males with specific expression in the reproductive system. Thus, *spo* is expressed during development and in the adult females, but not in adult males, where *spot* expression was detected. This shows complementary expression patterns of *spo* and *spot* during development and in the reproductive tissues of adult *Tribolium*. The finding that *spot* is specifically expressed in the male reproductive system which lacks *spo* expression, suggests that the expression patterns of these paralogous genes have diverged but are tightly regulated. *Shd* expression was not detected in the reproductive system of male *Tribolium,* presumably rendering this tissue incapable of producing 20E.

### The Enzymes Encoded by the *Tribolium* Halloween Genes Mediate Ecdysteroid Biosynthesis and are Essential for Development

**Figure 2:**
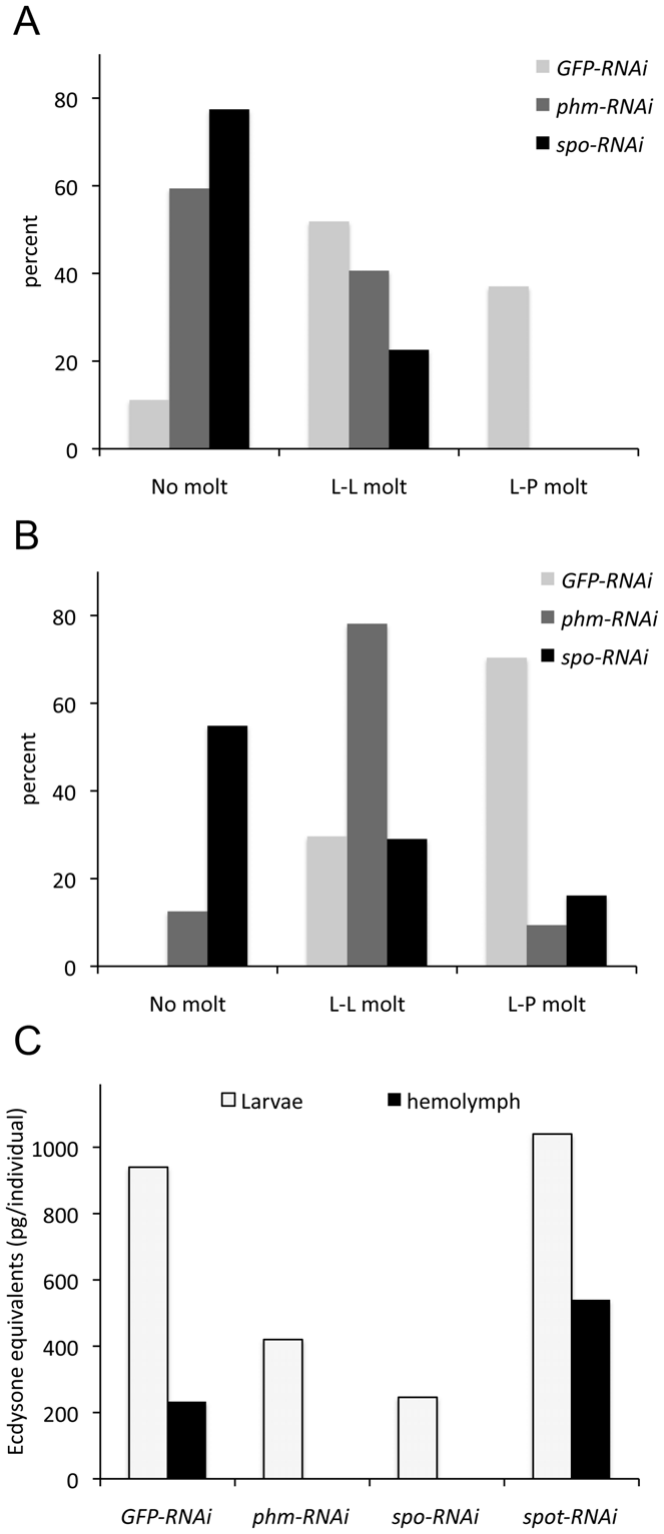
Knock down of *phm* and *spo* delays development and reduces ecdysteroid levels in *Tribolium*. Number of larvae that have undergone a molt after knock down of *phm* (*phm*-*RNAi*) and *spo* (*spo*-*RNAi*) expression 5 days (A) and 9 days (B) after injection with dsRNA. *GFP* dsRNA (*GFP-RNAi*) was used as a negative control. (C) Ecdysteroid levels in *spo-RNAi* and *phm-RNAi* larvae are reduced compared to the *GFP* control and to *spot-RNAi* 5 days after injection with dsRNA. Hemolymph ecdysteroid levels were below the limit of RIA detection (about 10 pg) in the hemolymph of *spo-RNAi* and *phm-RNAi* animals. L-L; larval-larval molt, L-P; larval-pupal molt.

To test whether the enzymes encoded by the Halloween genes have been functionally conserved in *Tribolium*, we chose to knock down the expression of *phm* and *spo* by injection of double stranded RNA (dsRNA) in the penultimate and final larval instars. As expected, the RNAi mediated reduction of *spo* and *phm* expression resulted in developmental arrest or delay (Figure 2). Five days after dsRNA injection, the majority of the *phm* or *spo* RNAi larvae had not molted and all larvae failed to pupate. For the *GFP-RNAi* control larvae, approximately 90% had undergone a molt five days after injection. Of these larvae approximately 50% had molted to another larval stage and 40% had pupated (Figure 2A). mRNA knockdown efficiencies were measured to be 78% for *phm* and 95% for *spo* (data not shown).

Nine days after dsRNA injection all control animals had molted and approximately 70% had formed pupae (Figure 2B). Although the majority of the *phm-RNAi* larvae had molted, only 10% had pupated. A stronger effect was observed in the case of the *spo-RNAi* animals where no ecdysis was observed in approximately 60% of the individuals and less than 20% had pupated 9 days after the dsRNA treatment. mRNA knockdown efficiencies were 92% for *phm* and 95% for *spo* nine days after injection of dsRNA (data not shown). In contrast, injecting dsRNA against *spot* did not affect larval molting and metamorphosis (data not shown). The data reveal that *spot* is not required for development during larval stages which is consistent with the expression data showing that the gene is not expressed during larval life. The data clearly demonstrate that the Halloween genes *spo* and *phm* are critical for the normal development of *Tribolium* and important for the molting process, which requires pulses of ecdysteroids.

To examine if knock down of *phm* and *spo* reduces ecdysteroid levels, we measured the ecdysteroid titers in the hemolymph and larval extracts (Figure 2C). The ecdysteroid titers were reduced in extracts from the *phm-RNAi* and *spo-RNAi* larvae, compared to the *GFP-RNAi* control animals, and were below the level of detection (approximately 10 pg) in the hemolymph of these animals. As expected, the *spot-RNAi* larvae showed no reduction in ecdysteroid levels compared to the control. These data show that Spo and Phm are important for ecdysteroid biosynthesis in *Tribolium*. *spot* is expressed specifically in the male accessory glands.

**Figure 3:**
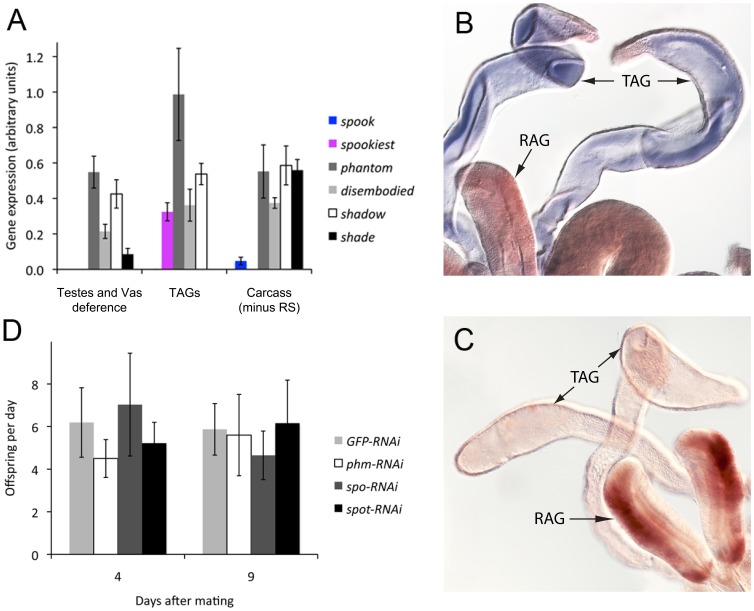
*Tribolium* male tubular accessory glands express the genes required for biosynthesis of E, but not 20E. (A) qPCR analysis shows that the tubular accessory glands express *spot* and the biosynthetic enzymes to produce E but not s*hd*, although E may be converted to 20E in other tissues as s*hd* is expressed in carcass (n = 3–4). (B) *In situ* hybridization with a *spot* antisense probe showing expression in the TAGs. (C) Negative control *in situ* hybridization using a *spot* sense probe. (D) The fertility of males with reduced expression of key E biosynthetic enzymes was examined by the number of progeny produced per day of virgin females mated with males injected with dsRNA (n = 6–7). Error bars are SE. RS; reproductive system, RAG; rod-shaped accessory gland, TAG; tubular accessory gland.

To further investigate possible ecdysteroidogenic tissues in males and the role of Spot, the TAGs were separated from the remaining parts of the reproductive system, including the testes, vas deferens and rod-shaped accessory glands, and Halloween gene expression was analyzed. Expression of *spot* was only observed in the TAGs and not in the remaining parts of the reproductive system. No expression was detected in the carcass of males after removal of the reproductive system (Figure 3A). Although very weak expression of *spo* was observed in the carcass, no expression was observed in the reproductive system. As Spo-like enzymes are essential for ecdysteroid production, these data indicate that the TAGs are the site of ecdysteroid production in the male reproductive system of *Tribolium*. The genes for the terminal hydroxylases, Phm, Dib and Sad, catalyzing the final three steps in the production of E were also expressed in the TAGs, but in other tissues as well. These observations suggest that some tissues have the biosynthetic capacity to perform late steps in the E synthetic pathway, but lack a Spo-like enzyme that is necessary for an upstream reaction and *de novo* synthesis. Expression of a *spo*-like gene may therefore distinguish ecdysteroidogenic tissues from non-ecdysteroidogenic tissues. Intriguingly, *shd* expression could not be detected in the TAGs, but the testes, vas deferens and carcass exhibited some expression.

The qPCR data were validated for *spot* by *in situ* hybridization and staining was observed specifically in the TAGs, and not in the remaining part of the reproductive system (Figure 3B and C), confirming the results obtained using qPCR.

### Reducing Expression of Ecdysteroidogenic Enzymes in Males does not Affect Fertility

We investigated the fertility in animals with reduced expression of *phm* and *spot* to investigate whether ecdysteroids are required for male reproduction. For this purpose, males injected with dsRNA after adult eclosion, were mated to virgin females. In these males, *phm* expression was reduced with 81%, whereas *spot* expression was reduced with 76%, and as much as 89% when the knockdown efficiency was measured specifically in the TAGs. The number of progeny from the male/female pairs with *phm-RNAi* and *spot-RNAi* males were not significantly reduced compared to the control (Figure 3D). Fertility of males with reduced *spo* expression was also analyzed as a control, since *spo* expression had not been detected in the reproductive system of adult males. As expected no reduction in fertility was observed.

### PTTH Influences Ecdysone Levels and likely Acts on Secondary Cells of the Accessory Glands of Male Drosophila

**Figure 4:**
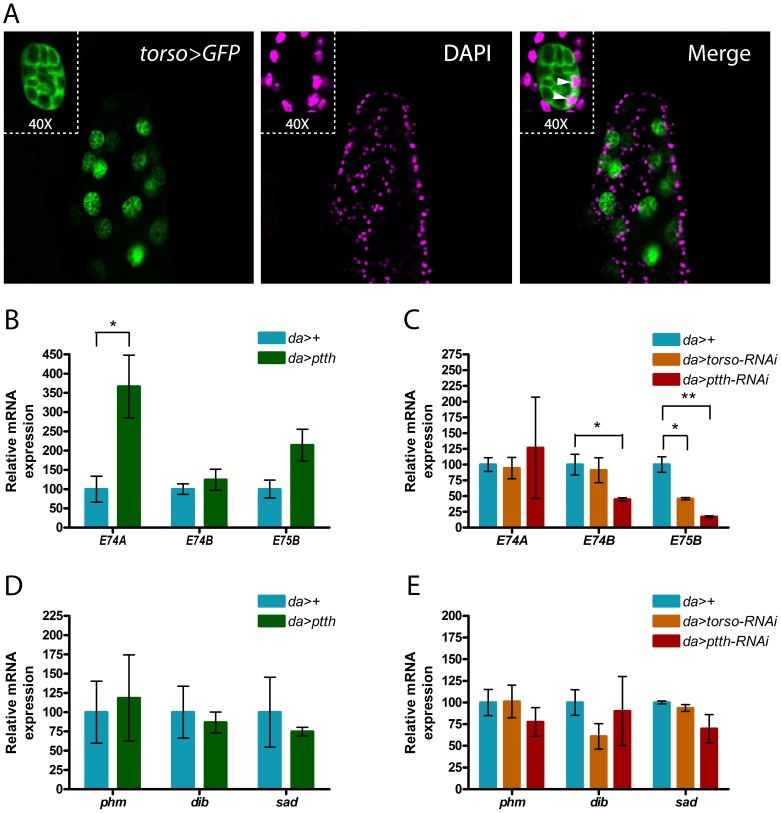
Adult ecdysteroid levels are regulated by PTTH that may target the accessory glands in *Drosophila*. (A) *GFP* expression driven by a *torso-Gal4* line in the bi-nucleated secondary cells of a male accessory gland of *Drosophila*. Inserts are magnifications showing the two nuclei of spherical secondary cells. Green: GFP, Magenta: DAPI. (B-E) Gene expression was analyzed by qPCR in adult *Drosophila* males overexpressing *ptth* (*da>ptth*) and in animals with reduced expression of *torso* (*da>torso-RNAi*) or *ptth* (*da>ptth-RNAi*). *da>+* (*da>* crossed to *w^1118^,* genetic background used by VDRC for generating the RNAi lines) was used as a control and expression was normalized to *rpL23* mRNA levels. (B and C) Ecdysteroid levels estimated as levels of expression of ecdysteroid target genes *E74A, E74B* and *E75B*. (D and E) Expression of Halloween genes *phm, dib* and *sad*.

As PTTH and Torso are involved in regulating the production of ecdysteroids during larval development [5], [29], [30], we suspected that they are also involved in the regulation of ecdysteroidogenesis in adult males. Since PTTH was not identified in Tribolium until very recently [31] and have not been functionally characterized, we turned to Drosophila, to identify possible PTTH target tissues. For this purpose the expression of torso was investigated using a torso-Gal4 line driving expression of UAS-GFP (Figure 4A). This line mimics endogenous expression of torso as GFP can be observed in the larval PG (data not shown). Interestingly, torso, as indicated by GFP, is expressed in large secondary bi-nucleated cells at the distal tip of the accessory gland lobes of male Drosophila. Together these data suggest that the accessory glands are not only a site of E synthesis, but also a possible target of PTTH signaling.

To investigate if PTTH regulates E synthesis in the adult, ptth, under control of UAS, was overexpressed using the ubiquitous daughterless-Gal4 (da>) driver. As a measure for ecdysteroid levels, expression of ecdysteroid target genes E74 (isoform A and B) and E75 (isoform B) was detected. E74A and E74B isoforms exhibit a dynamic response to changing levels of ecdysteroids [32]. E74B is induced by intermediate levels of ecdysteroids, but inhibited by higher levels required to induce the E74A isoform. Adult males overexpressing ptth (da>ptth) exhibited significantly elevated expression of E74A compared to the control (Figure 4B), whereas reducing expression of ptth with RNAi (da>ptth-RNAi) resulted in a significant decrease in the gene expression of E75B and E74B (Figure 4C). E74B expression was also reduced when the expression of torso was knocked down (da>torso-RNAi). These data demonstrate that PTTH is involved in regulating ecdysteroidogenesis in adult Drosophila males, possibly through Torso mediated signaling. No significant changes in expression of the Halloween genes phm, dib and sad were observed, neither in response to ptth overexpression nor to ptth or torso knock down (Figure 4 D and E).

## Discussion

It has been shown that the Halloween genes code for enzymes involved in ecdysteroidogenesis in several species of holometabolous insects [8], [10]–[12], [14]. Because these genes are required for 20E biosynthesis, they have been conserved in insects and perhaps most arthropods. So far, the only exception is the spider mite, *Tetranychus urticae*, which lacks *phm*, and thus, the ability to perform C25 hydroxylation of ecdysteroids [33]. Consequently, the main ecdysteroid is ponasterone A and not 20E in this species.

The Halloween genes are also found in *Tribolium*, but to our knowledge it has never been tested experimentally whether their function in ecdysteroidogenesis is conserved in this species. Our data show that expression of *spo, phm, dib, sad* and *shd* increases during the final larval instar which correlates with the increased ecdysteroid production necessary for molting. Reducing the expression of two of these genes, *phm* and *spo*, resulted in delayed molting or developmental arrest of *Tribolium* larvae, a phenotype typically observed in animals lacking ecdysteroids [5], [8], [29]. Consistent with this observation, ecdysteroid levels were low in these animals demonstrating that Spo and Phm are important for ecdysteroid biosynthesis during the larval stages. This correlation suggests strongly that the Halloween genes have been functionally conserved in *Tribolium.*

Although the four Halloween enzymes mediating the final hydroxylation steps in the biosynthesis of 20E have been identified, some upstream steps in the pathway remain less well characterized e.g. the Black Box [2], [8], [34]. Earlier studies have shown that the CYP307 family enzymes function in the Black Box, since supplying precursors of 20E downstream of the Black Box reaction rescues *Drosophila* larvae with reduced expression of *spok* [8], [35]. Moreover, these animals were not rescued by supplements of 7dC, a 20E precursor upstream of the Black Box reactions. The CYP307 paralogs are more highly conserved than the other Halloween P450 enzymes and are believed to have similar functions [36]. In support of this notion, ectopic expression of *spo*, rescues *Drosophila spok* mutants (Michael O’Connor, personal communication) and expression of *Bombyx mori spo* is sufficient to rescue *Drosophila spo* mutants [16]. *spo* and *spok* belong to the *CYP307A* subfamily. However, the genome of some insects, including *Anopheles*, *Apis* and *Tribolium*, contain a *CYP307B* subfamily gene [36]. Although subtle catalytic differences may exist between CYP307 enzymes, these conserved paralogs are likely to be functionally redundant products of gene duplications that occupy different spatio-temporal patterns of expression. Such a division of activity has been found in *Drosophila* where *spo* and *spok* exhibit different spatio-temporal expression patterns to support ecdysteroid biosynthesis in different tissues at distinct developmental stages [8]. The data support a similar scenario in *Tribolium* in which case *spo* is expressed during development and in the adult ovaries and *spot* is expressed in the reproductive system of adult males. In support of the data showing lack of *spot* expression during development, we confirmed that *spot* is not required to support ecdysteroid biosynthesis as the RNAi mediated knock down in the larval stages did not delay development.

This is, to our knowledge, the first data on the expression of a *CYP307B* family gene in any insect, suggesting that expression of these genes may be low and/or limited to a few cells in specific tissues, like the expression of *spot* in the TAGs of adult male *Tribolium*. As the genes encoding the terminal hydroxylases are conserved as 1∶1 orthologs, it is puzzling why the genes of the even more conserved *CYP307* family have been allowed to duplicate so that some species like *Tribolium* carry two paralogs. However, the available data suggest that these genes may have divided their effort to support E biosynthesis in a temporal and spatial restricted manner [15].

The highly specific expression of *spot* in the TAGs suggests that ecdysteroids are produced by the reproductive system of adult males. As *spot* seems to be the only CYP307 family member expressed at substantial levels in adult males, the TAGs may be the major site of ecdysteroidogenesis in adult males. Little is known about the function of steroid hormones in adult insects, but they are believed to be produced in the gonads, like the sex steroids of vertebrates, and it has previously been suggested that they might have a somewhat similar function [22]. In females of some insect species the ovaries produce ecdysteroids and their synthesis is required for oogenesis and the synthesis of vitellogenin [10]–[13], [19]–[21], [37]. In *Drosophila* mutants lacking the ability to synthesize 20E, egg development is arrested at stage 8–9 [8], [14]. The roles of ecdysteroids and their site of synthesis in males are much less clear. Ecdysteroids have been observed in testes and the accessory gland of male grasshoppers and in the testes of adult male blowflies [23], [24]. Recently Schwedes *et al.* [38] have documented the activity of the ecdysone receptor/ultraspiracle (EcR/USP) complex in numerous tissues in adult male *Drosophila,* suggesting that ecdysteroid signaling is important in adult male insects. High activity was observed in the male accessory gland, which could be explained by synthesis of ecdysteroids in this tissue.

Ecdysteroid levels in adult males are generally lower compared to the high-level pulses produced by the PG that drive transitions during development and those of female insects [22], [39]. This is consistent with our finding that Halloween gene expression in male adults is low compared to larvae, pupae and females. Biochemical identification of ecdysteroidogenic tissue has relied on the ability of tissues to convert labeled C into E and 20E. However, the low ecdysteroidogenic capacity of males makes it difficult to detect such conversions. Molecular approaches, such as those employed here, have provided some insights into the identification of ecdysteroidogenic tissues of the male mosquito, *Anopheles*. Using semi-quantitative PCR and *in situ* hybridization techniques it was shown that the genes coding for the terminal hydroxylases *phm*, *dib*, *sad* and *shd* are expressed specifically in the accessory glands of male *Anopheles*. In *Anopheles*, 20E produced in the accessory glands is stored and transferred to the female during mating [25], but the endocrine function of this 20E in males remains conjectural. As in *Tribolium*, expression of the genes coding for the biosynthetic enzymes necessary for ecdysteroid biosynthesis was not observed in the testes of *Anopheles*. However, whereas the testes of *Anopheles* seems to lack significant expression of all biosynthetic enzymes, *Tribolium* testes lack expression of a *CYP307* family gene which is indicating their inability to conduct *de novo* ecdysteroidogenesis. Although tissues lacking expression of a *spo*-like gene may be able to mediate downstream steps in the pathway, they are unlikely to have the capacity to synthesize E from C. Such capacity has been demonstrated in *Locusta migratoria* and *Manduca sexta* where biochemical analyses have shown that non-ecdysteroidogenic tissues, in addition to the PG, can convert the 5β-ketodiol, but not C, to E [26], [27]. These tissues presumably lack pathway activity upstream to the 5β-ketodiol, which is the ability to perform the Black Box reaction and possibly the C to 7dC conversion. Controlling the activity of *spo*-like genes makes sense as they participate in the possible rate-limiting black box step and expression of these genes may therefore be the determining factor for ecdysteroidogenesis. Expression of the downstream terminal hydroxylases may not require such a precise control since “leaky” expression of these genes alone will not enable a tissue to synthesize ecdysteroid *de novo*. Regulation of ecdysteroid biosynthesis at the level of Spo is also supported by evidence identifying *spo* as the only target of PTTH signaling in the E biosynthetic pathway in *Manduca* [40]. Consistent with this view, expression of *phm*, *dib*, *sad* and *shd* was detected outside the TAGs. Although it cannot be ruled out that tissues other than the TAGs in *Tribolium* synthesize ecdysteroids in adult males, the present data provides evidence that the TAGs are a major site.

**Figure 5:**
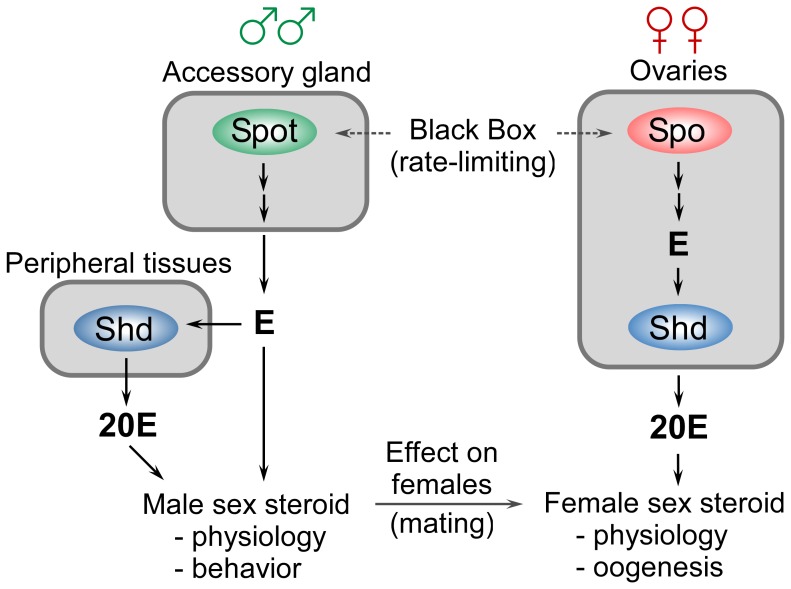
A model for sex-specific synthesis and action of ecdysteroids in adults. The ovaries of female *Tribolium* express *spo* and the genes for the terminal hydroxylases, including *shd*, required for synthesis of 20E. Male accessory glands also express a *spo*-like gene, *spot*, and the genes for the terminal hydroxylases required for synthesis of E, but not *shd*. However, s*hd* expression was detected in the carcass without the reproductive system indicating that E synthesized by the accessory gland might be converted to 20E in peripheral tissues, like during the larval stages. Alternatively, E produced by the accessory gland may be involved in male-specific hormone signaling or be transferred to females during mating. Multiple arrows indicate several steps in the biosynthetic pathway.

Intriguingly however, the TAGs seem to lack the expression of *shd,* making it likely that they synthesize E rather than 20E. This is similar to the larval PG that produces E which is released and converted to 20E in target tissues [2]. In contrast, all the genes necessary for the production of 20E, including *shd*, are expressed in the ovaries of *Tribolium*, as in *Drosophila*. Therefore it is likely that the ovaries primarily produce 20E, whereas the male reproductive system may synthesize E (Figure 5).

Although E is a precursor of 20E it induces a specific genetic response distinct from that of 20E in *Drosophila* larvae [41]. In support of an E-specific role, the distribution of E and 20E varies during pupal-adult development in *Manduca* [42]. During this stage a pulse of E precedes a pulse of 20E by several days, indicating that the two hormones have different functions. Another interesting observation is that E, but not 20E, induces vitellogenesis in the cockroach *Blaberus craniifer* [43]. This opens the possibility that E produced by males, and transferred to females during mating, is required to stimulate vitellogenesis. Thus, E produced in the accessory gland may affect female post-mating physiology and behavior as it has been suggested in *Anopheles* [25]. Alternatively, E synthesized in the accessory gland could be released into the hemolymph to affect male physiology and behavior. For example, a minor induction in the transcription of the male specific yellow protein in *Schistocerca gregaria* was observed in response to E, while 20E is an inhibitor, indicating that E and 20E might have sex-specific roles [44]. Some insight into the role of ecdysteroids in adult male insects comes from recent studies of *Drosophila*. The reduced ecdysteroid level of *DTS-3* mutants impairs memory formation in males [45]. Interestingly, ecdysteroid levels are significantly elevated in wild type males after courtship. Further, ecdysteroids affect sleep and longevity in adult *Drosophila* [46]–[48], providing evidence that they serve important physiological functions during adulthood in males as well as in females.

Ecdysteroids also influence male courtship behavior by regulation of the transcription factor *fruitless* [49]–[51]. A recent study also shows that conditional reduction of ecdysteroid signaling in the adult stage, and not during development, causes the males to display male-male courtship [52], indicating that the effects of ecdysteroids is not solely explained by neuronal wiring of a male-specific circuitry during development. This phenotype is also observed in males that lack PTTH [29], suggesting that PTTH may regulate E production in adults. Although PTTH was first purified from adult moths and is found in adult *Drosophila* [29], [53], its role in adults is not known. Here we present the first data showing that PTTH regulates ecdysteroid synthesis in adult insects, i.e. has functions other than eliciting and coordinating metamorphosis. Although PTTH influences the ecdysteroid levels, the data suggest that this effect does not require transcriptional regulation of the steroidogenic genes. Thus, PTTH probably mediates an acute response on steroidogenesis through post-transcriptional regulation in the adult, a scenario similar to that observed in the PG during development [30], [40]. The data indicating expression of the gene for the PTTH receptor Torso in the secondary cells of the accessory glands of male *Drosophila* provide molecular evidence that the accessory glands may be a target of PTTH. These data are supported by the recent identification of the accessory glands as the only PTTH-responsive tissue in adult *Manduca* [54], although no link was made between the PTTH-induced phosphorylation response in the accessory glands and ecdysteroidogenesis. Together with the reduced ecdysteroid levels observed in *ptth* and *torso* RNAi males, these data raise the possibility that PTTH regulates ecdysteroid production in the accessory glands.

Other peptide hormones, such as the insulin-like peptides, are known to be involved in the regulation of ecdysteroid signaling. In *Drosophila*, body size and developmental timing is influenced by the interplay between insulin and ecdysone signaling [55], [56]. Insulin-like peptides have been shown to stimulate ecdysteroid biosynthesis, and one specific insulin-like peptide, DILP8, may also be involved in suppressing ecdysteroid production to coordinate organ growth and maturation [57], [58]. In the mosquito *Aedes aegypti* and *Drosophila*, insulin has a stimulatory effect on ecdysteroidogenesis [59]–[62]. The fact that insulin stimulates ecdysteroid production in adults is interesting considering the potential role of ecdysteroids as sex-steroids. In crayfish, that also produces ecdysteroids, silencing of an insulin-like gene confers testicular degeneration and ovarian up regulation [63].

In several species ecdysteroids have been shown to influence spermatogenesis, by affecting the rate of mitosis and meiosis, and thus, differentiation of germ cells [37], [64]. Although ecdysteroids are required for female reproduction, male *Drosophila dib* mutants, which are unable to synthesize 20E, are fertile [65]. Similarly, we did not observe reduced production of offspring by *spot-RNAi* and *phm-RNAi Tribolium* males. It is possible that the knock down was insufficient to affect reproductive success. However, arguing against this possibility, injection of dsRNA into larvae efficiently reduced expression of *phm* and *spot* and the 20E production. One explanation is that E or 20E is not essential for male fertility. This also agrees with previous studies, finding that knock down of *phm* and *shd* does not affect fertility of male *Tribolium* [66], and that ecdysteroids are essential for female, but not male, germ cell development in *Drosophila* [67]. Interestingly, a recent study found that the secondary cells of the *Drosophila* accessory glands produce a substance that induces a female post-mating response by influencing egg laying activity and sexual receptivity [68]. Considering the evidence that E has been observed to be a more potent stimulator of vitellogenesis than 20E [43], one could speculate that E may be produced by the secondary cells in *Drosophila* males and passed to the female during mating.

As ecdysteroids are produced in the reproductive organs of adult insects, and in some species influences spermatogenesis, release of vitellogenin, memory and sex-specific physiology and behavior, they share several features with the vertebrate sex-hormones [22], [69]–[72]. In vertebrates, the reproductive system of males primarily synthesizes testosterone, the precursor of the female sex steroid, estradiol [69]. The conversion of testosterone to estradiol is catalyzed by a P450 enzyme, as Shd, that mediates the conversion of E to 20E in insects. Considering that E and 20E have distinct functions and have been proposed to function as sex steroids in insects [22], the present study provides the first evidence that males may synthesize E for sex-specific purposes whereas females presumably produce 20E.

## Materials and Methods

### Insects

*Tribolium castaneum* provided by Marek Jindra (Czech Academy of Science) were reared under constant temperature and 60% humidity under a 12∶12h light-dark cycle on whole wheat flour with 5% inactivated yeast. The following lines of *Drosophila* were used; *w^1118^* (VDRC), *NP3370-Gal4* (*torso-Gal4*) (a line with a Gal4 insertion in the *torso* enhancer: DGRC), *UAS-CD8-GFP* (Bloomington Stock Center), *daughterless-Gal4* (*da-Gal4*: Bloomington Stock Center), *UAS-ptth* (a gift from Michael O’Connor), *UAS*-*ptth-RNAi* #100502 (VDRC) and *UAS-torso-RNAi* #36280 (VDRC). All flies were reared under constant temperature, 60% humidity and with 12∶12h light-dark cycle on standard cornmeal.

### Quantitative PCR (qPCR)

Reproductive systems of male and female *Tribolium* were dissected in ice-cold insect saline [73]. Total RNA was isolated using RNeasy Mini kit (Qiagen) and treated with RNase-Free DNase to avoid genomic DNA contamination. For reproductive systems and TAGs, tissues pooled from 4–6 individuals were used and for each developmental stage RNA was extracted from four pooled animals. For measuring expression in adult *Drosophila* males, RNA was extracted from five animals 4–6 days after eclosion. RNA was quantified spectrophotometrically and cDNA was synthesized from oligo(dT) primers using standard procedures. Levels of transcripts were quantified using quantitative real time PCR (qPCR) with SYBR green as the reporter. Primers are given in Table S1 and qPCR conditions were: 95°C 10 min followed by 45 cycles of 95°C for 15 sec, 60°C for 15 sec and 72°C for 15 sec. Melting curve analysis was carried out for all PCR reactions to verify homogeneity of the PCR products. For the *spot* primers the PCR product was cloned into the pGEM Easy vector (Promega) and sequenced. As internal standards, ribosomal protein *rpS3* was used for *Tribolium* and ribosomal protein *rpL23* for *Drosophila*. As a control for contribution of genomic DNA contamination, non-reverse transcribed (NRT) controls were included.

### RNA Interference (RNAi) in *Tribolium*

For dsRNA synthesis, primers against specific regions of *spo*, *spot* and *phm* with minimal T7 polymerase promoter sequences at their 5′-ends were used (table S1). Primers were designed to generate approximately 400–750 bp PCR products with T7 promoters at both ends. PCR products for dsRNA synthesis were amplified from cDNA of male reproductive systems (*spot*) or prepupae (*spo* and *phm*). PCR conditions: 95°C 10 min followed by 10 cycles of 95°C for 30 sec, 55°C for 30 sec and 72°C for 1 min and 25 cycles of 95°C for 30 sec, 60°C for 30 sec and 72°C for 1 min and 72°C for 7 min. dsRNA was synthesized with the T7 polymerase using the MEGAscript RNAi kit (Ambion) according to the manufacturer’s protocol. Cold-anesthetized animals were aligned on a glass slide covered with double-sticky tape and injected with dsRNA (2 µg/µl) as described [74] until their abdomen was full and they had stretched visibly. A Transjector 5246 (Eppendorf) controlled by a Micromanipulator 5171 (Eppendorf) was used under a dissecting stereomicroscope. Injection with GFP dsRNA was used as a negative control. Larvae were injected into the dorsal side between the first and second abdominal segment. After eclosion injections into adult males were done in the abdominal body cavity laterally to avoid damaging genitals.

### Measuring Ecdysteroid Levels

For determination of ecdysteroid levels in dsRNA injected larvae, larval hemolymph was collected from 6 larvae by separation of the thorax and abdominal region in 0.75 ml insect saline [73]. Hemolymph was allowed to drain into the media for 5 min facilitated by a gentle pressure applied every minute. The incubation medium, containing the hemolymph, was then collected and the dissection dish washed with additional 0.5 ml insect saline to increase recovery of ecdysteroids. The medium was briefly centrifuged at 3000 rpm for 15 sec to sediment and remove traces of tissues. The remains of the animal were also collected for analysis. Ecdysteroid levels were determined by radioimmunoassay (RIA) as described [42].

### *In situ* Hybridization and Fluorescence Microscopy

*In situ* hybridization: The RNA sense probe for *in situ* hybridization was synthesized from linearized plasmid. For the antisense probe, primers against a specific region of *spot* with minimal T7 polymerase promoter sequences in the reverse primer (table S1) were used to generate a PCR-product from cDNA. DIG-labeled RNA probes were synthesized with the T7 polymerase and *in situ* hybridization was performed using standard methods. In brief, reproductive systems were dissected from adult *Tribolium* males, and fixed in 4% formaldehyde. Samples were proteinase K-treated, fixed again in 5% formaldehyde and prehybridized for 1–2 hrs at 55°C in hybridization solution (50% formamide, 5X SSC, 50 µg/ml heparin, 0.1% Tween 20, 100 µg/ml salmon sperm DNA). Hybridization with RNA-probes was performed over night at 55°C. Unbound probe was removed by extensive washing in hybridization solution at 55°C. Tissues were incubated at 4°C over night with an alkaline phosphatase conjugated anti-DIG antibody. Unbound antibody was removed and tissues were stained before mounting in 80% glycerol and 20% PBS. Fluorescence microscopy: Reproductive systems from *Drosophila* (*torso>GFP*) males were dissected and fixed in 4% formaldehyde. Tissues were stained with DAPI, mounted on glass slides in 80% glycerol and 20% PBT and analyzed using confocal microscopy (Zeiss LSM 710).

### Male Fertility

After eclosion males injected with dsRNA were removed from the slide and kept on whole wheat flour (5% yeast) in separate wells prior to mating. The fertility of injected males was checked by mating them to virgin females. Pupae were collected and separated by sexes to obtain virgin females. Four days after eclosion virgin females were mated to males 4 or 9 days after these were injected with dsRNA. Adults were removed from the flour two days after mating and the flour was incubated at 32°C for 4 weeks to check for number of offspring.

## Supporting Information

Figure S1 **Amino acid sequence conservation in the CYP307 family of ecdysteroidogenic enzymes.** (A) Neighbor joining tree illustrating the phylogenetic grouping of the CYP307 family into CYP307A and B subfamilies. (B) Alignment of the *Tribolium* CYP307A and B amino acid sequences with orthologs from *Anopheles, Drosophila, Bombyx,* and *Apis.* Conserved residues are shown in black boxes while grey shading denotes amino acids with similar properties.
(PDF)

Table S1 **Oligonucleotide primers.**
(PDF)
