# Supplementary figures and images for: Accessory Gland as a Site for Prothoracicotropic Hormone Controlled Ecdysone Synthesis in Adult Male Insects
Source: PLoS One. 2013 Feb 1;8(2):e55131. doi: 10.1371/journal.pone.0055131 (PMC3562185; doi:10.1371/journal.pone.0055131)

Figure S1

A

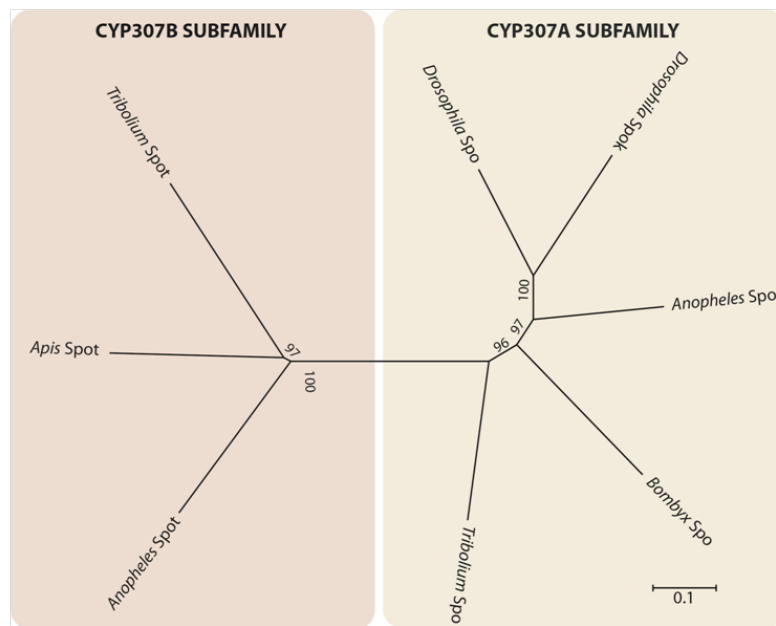

B

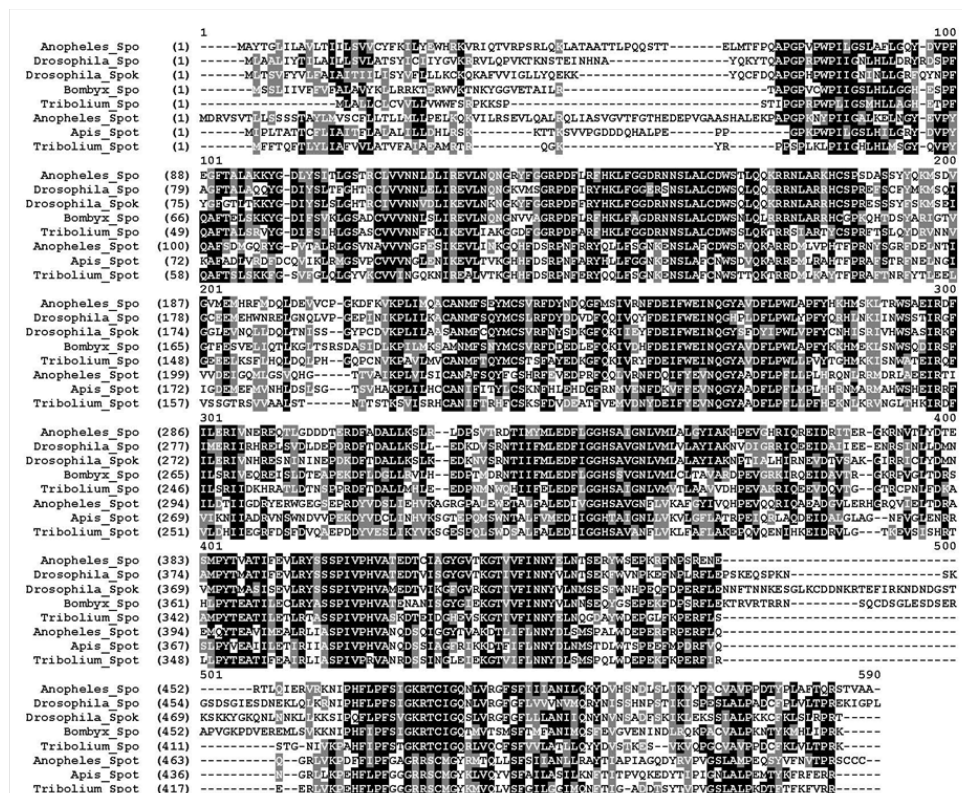

Supplement: Figure S1 — Amino acid sequence conservation in the CYP307 family of ecdysteroidogenic enzymes. (A) Neighbor joining tree illustrating the phylogenetic grouping of the CYP307 family into CYP307A and B subfamilies. (B) Alignment of the Tribolium CYP307A and B amino acid sequences with orthologs from Anopheles, Drosophila, Bombyx, and Apis. Conserved residues are shown in black boxes while grey shading denotes amino acids with similar properties. (PDF) [file pone.0055131.s001.pdf]
