# Supplementary material for: Accessory Gland as a Site for Prothoracicotropic Hormone Controlled Ecdysone Synthesis in Adult Male Insects
Source: PLoS One. 2013 Feb 1;8(2):e55131. doi: 10.1371/journal.pone.0055131 (PMC3562185; doi:10.1371/journal.pone.0055131)
Supplement: Table S1 — Oligonucleotide primers. (PDF) [file pone.0055131.s002.pdf]

Table S1
Oligonucleotide primers

**Primers**	**5´-3´ sequences**
**Primers used for qPCR**	
**In *Tribolium:***	
Tc*spo*/forward	GGGACGAGCCTGGACTGTT
Tc*spo*/reverse	CCCGTGCTGAAAGGAATGA
Tc*spot*/forward	CCTGAGCATTTCCTACCGTTC
Tc*spot*/reverse	CCCACCCAGAATCCCAAAG
Tc*phm*/forward	AAGAATGTGTGTCGGTGATGAA
Tc*phm*/reverse	TCGTGAGGTTTCGGAGTTAGTG
Tc*dib*/forward	ACAGGAAGAGCCACCTCACC
Tc*dib*/reverse	ACCATTCGGGTCCATTTGTT
Tc*sad*/forward	GCTAAGAGCCCGCAAATCC
Tc*sad*/reverse	GGTAAAGCCGCAAAGTCTCCT
Tc*shd*/forward	GGTCAACGAACAAGGTGAGG
Tc*shd*/reverse	GAGTCGGTCTGCGATGTAGTTT
Tc*rpS3*/forward	CAACTCGCACGGAAATCATC
Tc*rpS3*/reverse	CCACCTTCTCGCCATACAAA
**In *Drosophila:***	
*E74A/*forward	TTTCTCTGCCGTTGTCGTC
E74A/reverse	GCACTGAGACCCGCTCAC
*E74B/*forward	CGCGAGTTCAAAGTGCTCTA
E74B/reverse	GGAGGGAGAGTGGTGGTGT
*E75B/*forward	CAACAGCAACAACACCCAGA
E75B/reverse	CAGATCGGCACATGGCTTT
phm/forward	GGATTTCTTTCGGCGCGATGTG
phm/reverse	TGCCTCAGTATCGAAAAGCCGT
*dib/*forward	TGCCCTCAATCCCTATCTGGTC
dib/reverse	ACAGGGTCTTCACACCCATCTC
*sad/*forward	CCGCATTCAGCAGTCAGTGG
sad/reverse	ACCTGCCGTGTACAAGGAGAG
*rpL23/*forward	GACAACACCGGAGCCAAGAACC
rpL23/reverse	GTTTGCGCTGCCGAATAACCAC.

Primers used for dsRNA synthesis

Tc*spo*RNAi/forward	taatacgactcactatagggagaGCTCGCCCAGATTCACTTC
Tc*spo*RNAi/reverse	taatacgactcactatagggagaATGGTGGCTTCCGTGTAGG
Tc*spot*RNAi/forward	taatacgactcactatagggagaTTGTTGCCCTTCCACGAGA
Tc*spot*RNAi/reverse	taatacgactcactatagggagaCCCACCCAGAATCCCAAAG
Tc*phm*RNAi/forward	taatacgactcactatagggagaTCACGAGACGATGCCACTT
Tc*phm*RNAi/reverse	taatacgactcactatagggagaCCAATTCATCACCGACACAC
Tc*GFP*RNAi/forward	taatacgactcactatagggagaTTTTCACTGGAGTTGTCCCAAT
Tc*GFP*RNAi/reverse	taatacgactcactatagggagaTTTGTGTCCAAGAATGTTTCCA

Primers used for synthesis of the *in situ* probe

Tc*spot*/forward(2)	ACAGAAGAACATAAGGGAGGCG
Tc*spot*/reverse(2)	taatacgactcactatagggTCGAAGTCGTGTTGGTCG

T7 RNA polymerase promoter sites are shown in lower case letters. Tc: *Tribolium castaneum*
